# Neuronal seipin knockout facilitates Aβ-induced neuroinflammation and neurotoxicity via reduction of PPARγ in hippocampus of mouse

**DOI:** 10.1186/s12974-016-0598-3

**Published:** 2016-06-10

**Authors:** Yun Qian, Jun Yin, Juan Hong, Guoxi Li, Baofeng Zhang, George Liu, Qi Wan, Ling Chen

**Affiliations:** State Key Laboratory of Reproductive Medicine, Hanzhong Road 140, Nanjing, 210029 China; Department of Physiology, Nanjing Medical University, Hanzhong Road 140, Nanjing, 210029 China; Department of Neurology, First Affiliated Hospital of Nanjing Medical University, Guangzhou Road 300, Nanjing, 210029 China; Institute of Cardiovascular Sciences, Peking University and Key Laboratory of Cardiovascular Sciences, China Administration of Education, Beijing, 100191 China

**Keywords:** Seipin, β-amyloid (Aβ), Neuroinflammation, Peroxisome proliferator-activated receptor gamma (PPARγ), Glycogen synthase kinase-3 (GSK3)

## Abstract

**Background:**

A characteristic phenotype of congenital generalized lipodystrophy 2 (CGL2) that is caused by loss-of-function of seipin gene is mental retardation. Seipin is highly expressed in hippocampal pyramidal cells and astrocytes. Neuronal knockout of seipin in mice (*seipin*-KO mice) reduces the hippocampal peroxisome proliferator-activated receptor gamma (PPARγ) level without the loss of pyramidal cells. The down-regulation of PPARγ has gained increasing attention in neuroinflammation of Alzheimer’s disease (AD). Thus, the present study focused on exploring the influence of seipin depletion on β-amyloid (Aβ)-induced neuroinflammation and Aβ neurotoxicity.

**Methods:**

Adult male *seipin*-KO mice were treated with a single intracerebroventricular (i.c.v.) injection of Aβ_25–35_ (1.2 nmol/mouse) or Aβ_1–42_ (0.1 nmol/mouse), generally a non-neurotoxic dose in wild-type (WT) mice. Spatial cognitive behaviors were assessed by Morris water maze and Y-maze tests, and hippocampal CA1 pyramidal cells and inflammatory responses were examined.

**Results:**

The Aβ_25–35/1–42_ injection in the *seipin*-KO mice caused approximately 30–35 % death of pyramidal cells and production of Hoechst-positive cells with the impairment of spatial memory. In comparison with the WT mice, the number of astrocytes and microglia in the *seipin*-KO mice had no significant difference, whereas the levels of IL-6 and TNF-α were slightly increased. Similarly, the Aβ_25–35/1–42_ injection in the *seipin*-KO mice rather than the WT mice could stimulate the activation of astrocytes or microglia and further elevated the levels of IL-6 and TNF-α. Treatment of the *seipin*-KO mice with the PPARγ agonist rosiglitazone (rosi) could prevent Aβ_25–35/1–42_-induced neuroinflammation and neurotoxicity, which was blocked by the PPARγ antagonist GW9962. In the *seipin*-KO mice, the level of glycogen synthase kinase-3β (GSK3β) phosphorylation at Tyr216 was elevated, while at Ser9, it was reduced compared to the WT mice, which were corrected by the rosi treatment but were unaffected by the Aβ_25–35_ injection.

**Conclusions:**

Seipin deficiency in astrocytes increases GSK3β activity and levels of IL-6 and TNF-α through reducing PPARγ, which can facilitate Aβ_25–35/1–42_-induced neuroinflammation to cause the death of neuronal cells and cognitive deficits.

**Electronic supplementary material:**

The online version of this article (doi:10.1186/s12974-016-0598-3) contains supplementary material, which is available to authorized users.

## Background

Congenital generalized lipodystrophy (CGL) is an autosomal recessive disorder that is characterized by a near-total loss of adipose tissue [1]. Genome-wide linkage analysis has identified two loci related to CGL, i.e., CGL1 mutations in the 1-acylglycerol-3-phosphate O-acyl transferase 2 (AGPAT2) gene and CGL2 mutations in the Berardinelli-Seip congenital lipodystrophy 2 (BSCL2) gene that encodes seipin [2]. CGL2 patients with loss-of-function mutations in the seipin gene exhibit much higher rates of mental retardation than CGL1 patients [3, 4]. Seipin is highly expressed in the neuronal cells of the cortex, cerebellum, hippocampus, and hypothalamus [5, 6]. The seipin knockout in rats or mice causes spatial cognitive deficits through the synaptic dysfunction in hippocampal CA1 regions without loss of pyramidal cells [5, 7].

Seipin, an exclusive endoplasmic reticulum-residing N-glycosylated protein, can affect the generation of peroxisome proliferator-activated receptor gamma (PPARγ) [8]. The level of PPARγ is reduced in the embryonic fibroblasts of seipin-deficient mice [9]. We have recently reported that the neuronal knockout of seipin in mice (*seipin*-KO mice) reduces the expression of the hippocampal PPARγ [7, 10, 11]. The PPARγ is expressed in astrocytes and microglia and exerts an anti-inflammatory effect [12]. PPARγ agonists can inhibit the activations of microglia and astrocytes [13] and reduce the production of pro-inflammatory cytokines [14]. PPARγ deficiency has been reported to increase the neuroinflammation in allergic encephalomyelitis [15] and multiple sclerosis [16]. The levels of pro-inflammatory cytokines, such as tumor necrosis factor-α (TNF-α), interleukin-6 (IL-6), are elevated in Alzheimer’s disease (AD) brains [17]. The activation of PPARγ can enhance the cognitive reserve in humans with AD and in mouse model for AD amyloidosis [18]. The activation of PPARγ can improve hippocampus-dependent cognitive deficits in AD mouse models [19]. The PPARγ activation has been recently shown to mitigate the neuronal inflammation in chronic and acute neurological insults [20]. The PPARγ has received increasing attention in AD due to its anti-inflammatory function [21]. The hippocampal astrocytes express the seipin protein [10]. Thus, it should be interesting to examine whether seipin deficiency in astrocytes through reducing PPARγ affects Aβ-induced neuroinflammation and neurotoxicity.

The beneficial effects of PPARγ ligands potentially involve the suppression of signal transducer and activator of transcription (STAT) [22]. Li et al. have reported the elevation of STAT3 activity in the hippocampus of *seipin-*KO mice [10]. The STAT family of transcription factors has a central role in inflammatory reactions through regulating the expressions of multiple cytokines. In addition, the activation of STAT3 is known to be crucial for the differentiation of astrocytes [23]. Adult *seipin-*KO mice showed an increase in the astrocytic differentiation of progenitor cells in the hippocampal dentate gyrus, which can be corrected by the activation of PPARγ [10]. The STAT3 activation is highly dependent on the phosphorylation of glycogen synthase kinase-3 (GSK3) [24]. GSK3 is a constitutively active Ser/Thr kinase consisting of GSK3α and GSK3β isoforms. GSK3β has been identified as a strong promoter of pro-inflammatory cytokines, including TNF-α and IL-6 [25, 26].

Among the Aβ fragments, a peptide bearing 11 amino acids (25–35) (Aβ_25–35_) is the shortest fragment of Aβ that is processed in vivo by brain proteases [27]. This peptide retains the ability to self-aggregate and elicits essentially the same toxicity as the full-length peptide despite lacking the hydrophobic C terminal sequence of five amino acids that is present in Aβ_1–40_ [28]. In this study, we treated the adult male *seipin*-KO mice with a single i.c.v. injection of Aβ_25–35_ at a non-neurotoxic dose in control mice [29, 30], and then observed the Aβ_25–35_ neurotoxicity (the death of the hippocampal pyramidal cells) and Aβ_25–35_-induced neuroinflammation (the activation of astrocytes and microglia, the levels of IL-6 and TNF-α). To test the involvement of reduced PPARγ by seipin deficiency, we investigated the effects of the PPARγ agonist and the PPARγ antagonist on Aβ_25–35_-induced neuroinflammation and Aβ_25–35_ neurotoxicity in the *seipin*-KO mice. The results of the present study indicate that seipin deficiency in astrocytes facilitates the Aβ_25–35_-induced neuroinflammation through reducing PPARγ to elevate GSK3β activity, which causes the death of pyramidal cells leading to the spatial cognitive deficits.

## Methods

### Generation of seipin knockout (*seipin*-KO) mice

All animal handling procedures followed the guidelines for Laboratory Animal Research of Nanjing Medical University. The use of animals was approved by the Institutional Animal Care and Use Committee of Nanjing Medical University. The mice were maintained in constant environmental conditions (temperature 23 ± 2 °C, humidity 55 ± 5 %, and a 12:12-h light/dark cycle). The neuronal *seipin*-KO mice were generated as described elsewhere [11]. The genotypes of the *seipin*-KO mice were identified by PCR using genomic DNA from their tails. The DNA was amplified with the following primers: 5′-CTTGTCTCAAAGGGGTCT-3′ (forward primer for loxP) and 5′-TCAACAGAACAGACGCT-3′ (reverse primer for loxP); and 5′-GCGGTCTGGCAGTAAAAACTATC-3′ (forward primer for nestin-Cre) and 5′-GTGAAACAGCATTGCTGTCACTT-3′ (reverse primer for nestin-Cre). All animals received a standard laboratory diet before and after all procedures. In this study, 16-week-old male *seipin*-KO mice (*n* = 88) and wild-type (WT) mice (*n* = 56) were used at the beginning of all experiments. All mice were randomly assigned to one of the following six experimental groups: WT mice (*n* = 24), Aβ_25–35_-treated WT mice (Aβ_25–35_-mice, *n* = 16), Aβ_1–42_-treated WT mice (Aβ_1–42_-mice, *n* = 16), *seipin*-KO mice (*n* = 32), Aβ_25–35_-treated *seipin*-KO mice (Aβ_25–35_-KO mice, *n* = 40), and Aβ_1–42_-treated *seipin*-KO mice (Aβ_1–42_-KO mice, *n* = 16). Each experiment was performed by two experimenters who were blinded to the experimental groups. The behavioral tests (*n* = 16 mice for each group) and histological observations (*n* = 8 mice for each group) or Western blot analyses (*n* = 8 mice for each group) were sequentially performed in the same cohorts.

### Injection (i.c.v.) of Aβ_25–35_ and Aβ_1–42_

The Aβ_25–35_ (Sigma, St. Louis, MO, USA) was aggregated by incubation in distilled water (1 mg/ml) at 37 °C for 4 days and diluted to the final concentration with saline immediately before the experiment [30]. After this treatment, two types of birefringent fibril-like structures and globular aggregates of Aβ_25–35_ can be observed by light microscopic observation [30]. The Aβ_1–42_ (Sigma, St. Louis, MO, USA) was dissolved in 1,1,1,3,3,3-hexafluoro-2-propanol (HFIP, Sigma-Aldrich) and then flash-freezed in liquid nitrogen and lyophilized to completely remove the solvent [31]. Lyophilized Aβ_1–42_ was dissolved in 100 mM NaOH at a concentration of 6 mg/ml, aliquoted in 50 μl volumes [32]. The mice were anesthetized with ketamine (100 mg/kg, i.p.) and xylazine (10 mg/kg, i.p.) [33] and placed in a stereotactic apparatus (Motorized Stereotaxic StereoDrive; Neurostar). The “aggregated” Aβ_25–35_ (1.2 nmol/mouse at 2 μl) or the Aβ_1–42_ (0.1 nmol/2 μl in 0.1 M phosphate-buffered saline) was injected into the ventricles (0.3 mm posterior, 1.0 mm lateral, and 2.5 mm ventral to the bregma) using a stepper-motorized micro-syringe at a rate of 0.3 μl/min [34]. The injection site was confirmed by the injection of Indian ink in the preliminary experiments. The mice infused with the same volume of vehicle served as the control group.

### Drug administration

The PPARγ agonist rosiglitazone (rosi; Enzo, Farmingdale, NY) and the PPARγ antagonist GW9962 (Sigma, St. Louis, MO, USA) were dissolved in dimethyl sulfoxide (DMSO) and then diluted in 0.9 % saline to a final concentration of 0.5 % DMSO. The oral administration (p.o.) of rosi was given daily at a dose of 5 mg/kg [7]. The GW9962 (1 mg/kg/day) was injected intraperitoneally (i.p.) [35].

### Behavioral examination

The behavioral performances on days 5–14 after Aβ_25–35_ injection were captured via video recording (Winfast PVR; Leadtek Research, Fremont, CA) and analyzed using TopScan Lite 2.0 (Clever Sys., Reston, VA). *Morris water maze task*: The water maze task was consecutively performed to examine the spatial memory [29]. A pool (diameter = 120 cm) made of black-colored plastic was prepared. The water temperature was maintained at 20 ± 1 °C with a bath heater that was used before the sessions. On days 1–2 of training, a cylindrical black-colored platform (diameter = 7 cm) was placed 0.5 cm above the surface of the water. A mouse was randomly released into one of the four different quadrants and allowed to swim for 90 s. The latency to reach the visible platform was measured. On days 3–7 of the training, the platform was moved to the opposite quadrant of the previously visible platform and submerged 1 cm below the surface of the water. Four trials were conducted each day with an intertrial interval of 30 min. The average swimming speeds (m/s) and latencies (s) to reach the platform were scored for all trials. If the mouse was unable to reach the platform within 90 s, the experimenters gently assisted it onto the platform and allowed it to remain there for 15 s. Each mouse began in one of the four quadrants, which was selected in a random manner. On day 8 of the training, a probe trial was performed by removing the platform. The mouse was released from the opposite quadrant relative to the previous location of the platform and allowed to swim for 90 s. The percentages of swimming time spent in the target quadrant, opposite quadrant, and right and left adjacent quadrants were determined. *Y-maze task*: Spatial working memory performance was assessed by recording spontaneous alternation behavior in a Y-maze [30]. A Y-maze task was performed 48 h after the probe trial task. The Y-maze was constructed of black-painted wood. Each arm was 40-cm long, 13-cm high, 3-cm wide at the bottom, and 10-cm wide at the top, and the arms converged at equal angles. Each mouse was placed at the end of one arm and allowed to move freely through the maze during an 8-min session. The series of arm entries were recorded visually, and arm entries were considered to be completed when the hind paws of the mouse were completely placed in the arm. Alternations were defined as successive entries into the three arms on overlapping triplet sets. The percentage of alternations was calculated as the ratio of actual to possible alternations (defined as the total number of arm entries minus two). The scorers were blinded to the treatment groups.

### Histological examination

The mice were anesthetized with chloral hydrate (400 mg/kg, i.p.) and perfused with 4 % paraformaldehyde. The brains were removed and immersed in 4 % paraformaldehyde at 4 °C overnight.

*Toluidine blue staining*: The brains were processed for paraffin embedding, and coronal sections (5 μm) were cut. The pyramidal cells in the hippocampal CA1 region were identified using a conventional light microscope (Olympus DP70, Tokyo, Japan) with a 60× objective. The healthy pyramidal neurons exhibited round cell bodies with plainly stained nuclei. *Stereological counting CA1 pyramidal cells*: The brains were transferred into 30 % sucrose. After gradient dehydration, the coronal sections of the hippocampus (40 μm) were cut with a freezing microtome (Leica, Nussloch, Germany) and stained with toluidine blue. Every fourth section was obtained for consecutive cell quantification analyses. Stereological cell counting was performed by a stereological system, consisting of a light microscope with a CCD camera (Olympus DP70), a motorized specimen stage for automatic sampling (MicroBrightField, Williston, USA), and a computer running Microbrightfield Stereo Investigator software (Microbrightfield, Williston, VT, USA) [36]. All counts were performed by the same investigator, who was blind to the treatment. The total numbers of healthy pyramidal cells throughout the hippocampal CA1 region were counted using the optical fractionators’ method. The section thickness was measured using a dissector height of 10 μm. The layer of pyramidal cells was defined according to the terminology of Blackstad [37]. The boundary between the CA3 and CA1 subregions was identified by the differential thickness of the cell layers and by the smaller cell somas of the CA1 subregion. The boundary between CA1 and the subiculum was defined as the line separating the contiguous cells of CA1 from the more widely spaced cells of the subiculum.

*Hoechst staining*: Coronal paraffin sections (5 μm) were incubated in Hoechst 33342 (1 μg/ml, Cell Signaling Technology, Inc., Boston, MA, USA) for 2 min. *Quantitative analyses of Hoechst-positive cells*: Hoechst-positive (Hoechst^+^) cells in hippocampal CA1 region were observed by a conventional light microscope (Olympus DP70, Japan) with 60× objective and counted. The density of Hoechst^+^ cells was expressed as the number of cells per millimeter length along the pyramidal cells layer.

*GFAP and Iba1 staining*: The hippocampal sections (40 μm) were cut using a cryostat. Every fourth section was obtained with a randomly chosen starting section. The free-floating sections were blocked with 3 % normal goat serum for 60 min. Glial fibrillary acidic protein (GFAP) and ionized calcium-binding adapter molecule 1 (Iba1) immuno-staining were performed using the following primary antibodies and incubation at 4 °C overnight: mouse anti-GFAP antibody (1:500; Millipore, Billerican, MA, USA) and goat polyclonal anti-Iba1 antibody (1:500; Abcam, Cambridge, UK). Then, the sections were incubated in biotin-labeled goat anti-mouse IgG antibody (1:400; Santa Cruz Biotechnology, Santa Cruz, CA, USA) or biotin-labeled rabbit anti-goat IgG antibody (1:500; Bioworld Technology, Inc., St. Louis Park, MN, USA) for 2 h at room temperature. Immuno-reactivity was visualized based on the avidin-biotin-horseradish peroxidase complex (ABC Elite; Vector Laboratories, Inc., Burlingame, CA, USA).

*Counting GFAP- and Iba1-positive cells in the hippocampal CA1*: The GFAP-positive (GFAP^+^) cells and Iba1-positive (Iba1^+^) cells in the hippocampal CA1 radiatum layer were counted using a conventional light microscope (Olympus DP70). The radiatum layer was outlined at ×5 magnification. Systematic random sampling was achieved with a uniform sampling grid superimposed randomly over the region. A counting frame was placed in each square of the grid, and the cells were counted within that frame at ×40 magnification. A cell was counted if the nucleus was stained and was (a) in focus, (b) within the counting frame or crossed the green counting frame inclusion line, and (c) within the optical disector. Total estimated cell number per section = number of counted cells × (1/ssf) × (1/asf) × (1/hsf), where ssf is the section sampling fraction, asf is the area sampling fraction, and hsf is the height sampling fraction [38]. The densities of GFAP^+^ cells and Iba1^+^ cells are expressed as the mean numbers per mm^3^, which were normalized to the control value obtained from WT mice [39].

*Seipin/GFAP or Iba1 double immuno-staining*: The coronal sections (5 μm in thickness) were placed in gelatine-coated slides, blocked with 3 % normal goat serum, and then incubated in rabbit anti-seipin antibody (1:200; Abcam) at 4 °C overnight, which was revealed using CY3-labeled anti-rabbit IgG antibody (1:200; Millipore) or FITC-labeled anti-rabbit antibody (1:200; Millipore) and mouse monoclonal anti-GFAP antibody (1:200; Millipore) or goat polyclonal anti-Iba1 antibody (1:500; Abcam), which was revealed using FITC-labeled anti-mouse antibody (1:50; Millipore) or CY3-labeled anti-goat IgG antibody (1:200; Millipore). The immune-positive cells/fibers were observed by a confocal laser-scanning microscope (Leica, Heidelberg, Germany).

### Western blot analyses

The mice were decapitated under deep anesthesia with chloral hydrate. The hippocampus was quickly removed and homogenized in a lysis buffer containing 50 mM Tris-HCl (pH 7.5), 150 mM NaCl, 5 mM EDTA, 10 mM NaF, 1 mM sodium orthovanadate, 1 % Triton X-100, 0.5 % sodium deoxycholate, 1 mM phenyl-methylsulfonyl fluoride, and a protease inhibitor cocktail (Complete; Roche, Mannheim, Germany). The protein concentration was determined with a BCA Protein Assay Kit (Pierce Biotechnology, Rockford, IL, USA). The total proteins (20 μg) were separated by SDS-poly-acrylamide gel electrophoresis (SDS-PAGE) and transferred to a polyvinylidene difluoride membrane. The membranes were incubated with 5 % nonfat dried milk in Tris-buffered saline containing 0.1 % Tween 20 for 60 min at room temperature and were then incubated with a mouse monoclonal anti-phospho-GSK3β (Tyr216) antibody (1:1000; Cell Signaling Technology), a rabbit monoclonal anti-phospho-GSK3β (Ser9) antibody, and an anti-phospho-STAT3 antibody (1:1000; Cell Signaling Technology); a rabbit polyclonal anti-IL-6 antibody (1:1000; Abcam), a mouse monoclonal anti-TNF-α antibody and a PPARγ antibody (1:200; Santa Cruz Biotechnology); or a mouse monoclonal anti-β-actin antibody (1:1000; Cell Signaling Technology) and a rabbit monoclonal anti-GAPDH antibody (1:1000; Cell Signaling Technology) at 4 °C overnight. The membranes were then incubated with HRP-labeled secondary antibodies (1:10,000; Millipore). The blots were stripped by incubation in a stripping buffer (Restore, Pierce) for 5 min, incubated with rabbit polyclonal antibodies for GSK3β or STAT3 (1:1000; Cell Signaling Technology) and developed using an ECL Detection Kit (Millipore). The Western blot bands were scanned and analyzed with an image analysis software package (ImageJ; NIH Image, Bethesda, MD, USA).

### Data analysis/statistics

The data were retrieved and processed with the Microcal Origin 8.0 software. The group data are expressed as the means ± standard errors, and significance was tested with Student’s *t* tests or ANOVAs with or without repeated measures, followed by Bonferroni post hoc analysis for multiple comparisons. The results of the Morris water maze test were analyzed using repeated-measures ANOVA. The statistical analyses were performed using Stata 7 software (STATA Corporation, College Station, TX, USA). Differences were considered statistically significant at *P* < 0.05.

## Results

### Seipin deficiency enhances Aβ_25–35_-induced cognitive deficits

**Fig. 1:**
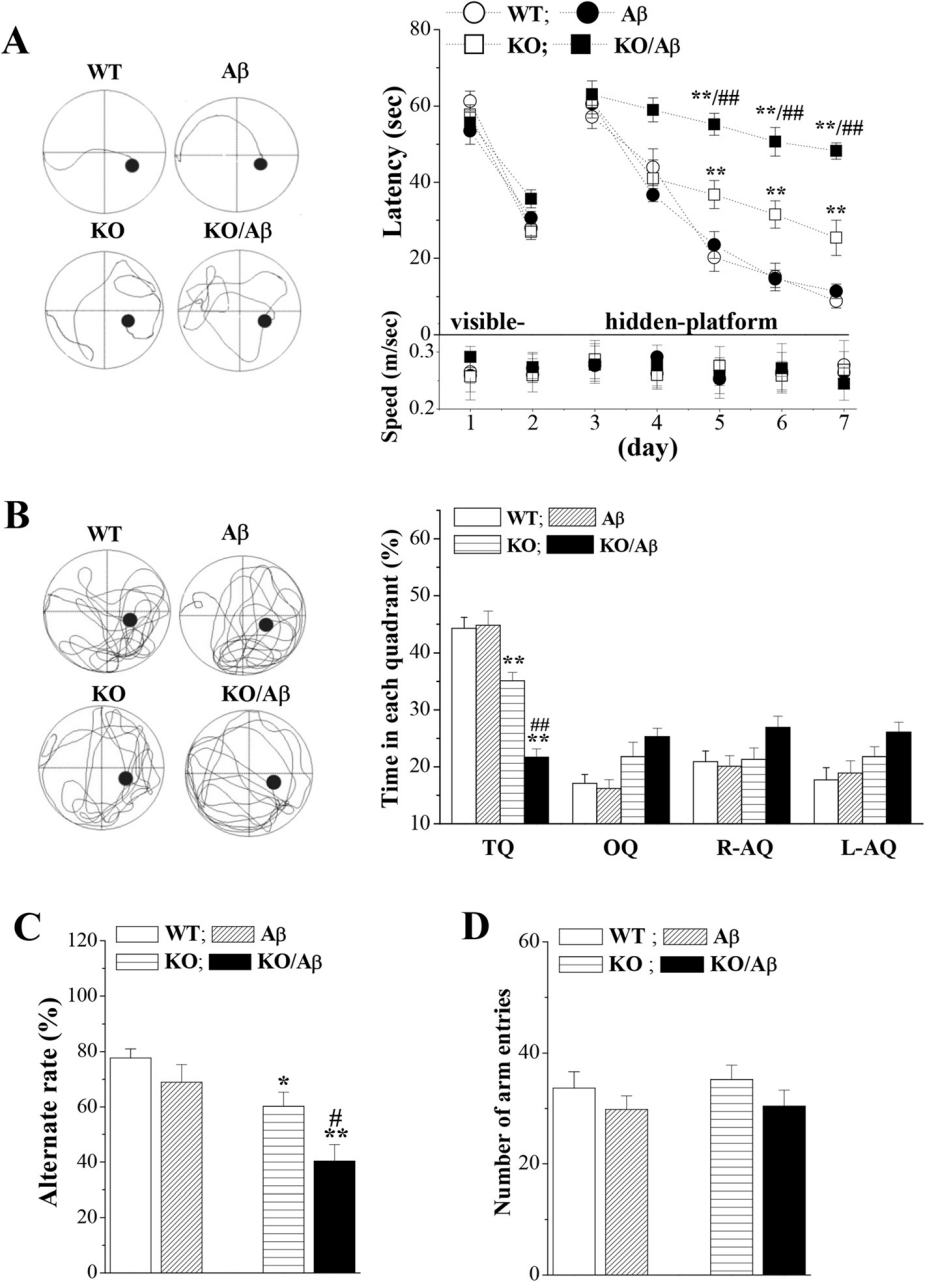
Seipin deficiency enhances Aβ_25–35_-induced cognitive deficits. **a** The latencies (s) to reach the visible and hidden platforms (*upper panel*) and the swimming speeds (m/s, *bottom panel*) of WT mice (WT), Aβ_25–35_ mice (Aβ), *seipin*-KO mice (KO), and Aβ_25–35_-KO mice (KO/Aβ) in the Morris water maze (MWM) test. The *left panels* illustrate representative swimming tracks of the mice searching for the underwater platform on day 5 after training. The *black dots* indicate the position of the hidden platform. **P < 0.01 vs. WT mice; ^##^ *P* < 0.01 vs. *seipin*-KO mice (repeated-measures ANOVA with Bonferroni’s test). **b** The *bars* represent the percentages of time spent in the target quadrant (TQ), opposite quadrant (OQ), and right (R) and left (L) adjacent quadrants (AQ) of the probe test. The *left panels* illustrate typical tracks. **P < 0.01 vs. WT mice; ^##^ *P* < 0.01 vs. *seipin*-KO mice (two-way ANOVA with Bonferroni’s test). **c**, **d** The *bar graphs* display group means in the alternation rate (%) and the numbers of arm entries (8 min) in the Y-maze (Y-M) task. *P < 0.05 and **P < 0.01 vs. WT mice; ^#^ *P* < 0.05 vs. *seipin*-KO mice (two-way ANOVA with Bonferroni’s test)

Spatial memory was examined using place learning in a Morris water maze task from day 5 after the injection (i.c.v.) of Aβ_25–35_. The mean latency required to find the visible platform on days 1–2 of training and the subsequent escape latencies to reach the hidden platform on days 3–7 of training are illustrated in Fig. 1a. First, the latency to reach the visible platform was not affected by genotype (*F*_(1, 60)_ = 0.179, *P* = 0.674), Aβ_25-35_ injection (*F*_(1, 60)_ = 0.054, *P* = 0.818), or the genotype × Aβ_25–35_ interaction (*F*_(1, 60)_ = 3.159, *P* = 0.081). Subsequently, repeated-measures ANOVA revealed that the escape latency to reach the hidden platform progressively decreased with training days in all groups (*F*_(4, 240)_ = 71.948, *P* < 0.001), and this effect was influenced by genotype (*F*_(1, 60)_ = 190.133, *P* < 0.001), Aβ_25–35_ (*F*_(1, 60)_ = 39.457, *P* < 0.001), and the genotype × Aβ_25–35_ interaction (*F*_(1, 60)_ = 36.701, *P* < 0.001). The *seipin*-KO mice required longer times to reach the hidden platform on days 5–7 of training than did the WT mice (*P* < 0.01). Furthermore, the Aβ_25–35_-injected (1.2 nmol/mouse) *seipin*-KO mice exhibited a significantly increased escape latency to the hidden platform (Aβ_25–35_-KO mice, *P* < 0.01) compared with the *seipin*-KO mice, but this effect was not observed in the Aβ_25–35_-injected WT mice (Aβ_25–35_-mice, *P* > 0.05) relative to the non-injected WT mice. However, the mean swimming speeds during the visible platform and hidden platform tests exhibited by Aβ_25–35_-mice (0.26 ± 0.01 m/s, *P* > 0.05), *seipin*-KO mice (0.27 ± 0.03 m/s, *P* > 0.05), and Aβ_25–35_-KO mice (0.27 ± 0.04 m/s, *P* > 0.05) were not significantly different from those of the WT mice (0.26 ± 0.03 m/s).

A probe test was performed 24 h after the hidden platform test to measure the strength of the memory trace. The swimming times spent in the target quadrant, opposite quadrant, and right and left adjacent quadrants are showed in Fig. 1b. The main effect of genotype (*F*_(1, 60)_ = 74.061, *P* < 0.001) and Aβ_25–35_ (*F*_(1, 60)_ = 11.813, *P* = 0.001) were that the *seipin*-KO mice spent less swimming time in the target quadrant than the WT mice (*P* < 0.01). Furthermore, there was a significant interaction of genotype × Aβ_25–35_ (*F*_(1, 60)_ = 13.716, *P* < 0.001). Notably, the swimming time in the target quadrant of the Aβ_25–35_-KO mice was shorter than that of the *seipin*-KO mice (*P* < 0.01), whereas the Aβ_25–35_-WT mice exhibited no significant difference compared with the WT mice (*P* > 0.05).

Spatial working memory performance was assessed with a Y-maze task. There was a main effect of genotype on the alternation ratio (*F*_(1, 60)_ = 18.472, *P* < 0.001; Fig. 1c), but no effect on the number of arm entries was observed (*F*_(1, 60)_ = 0.165, *P* = 0.686; Fig. 1d). The alternation ratio of the *seipin*-KO mice was reduced compared to that of the WT mice (*P* < 0.05). Additionally, there was a significant interaction of genotype × Aβ_25–35_ for the alternation ratio (*F*_(1, 60)_ = 7.214, *P* = 0.009). Notably, Aβ_25–35_-KO mice exhibited a significant reduction in the alternation ratio compared with the *seipin*-KO mice (*P* < 0.05).

### Activation of PPARγ prevents Aβ_25–35_-induced cognitive deficits in *seipin*-KO mice

**Fig. 2:**
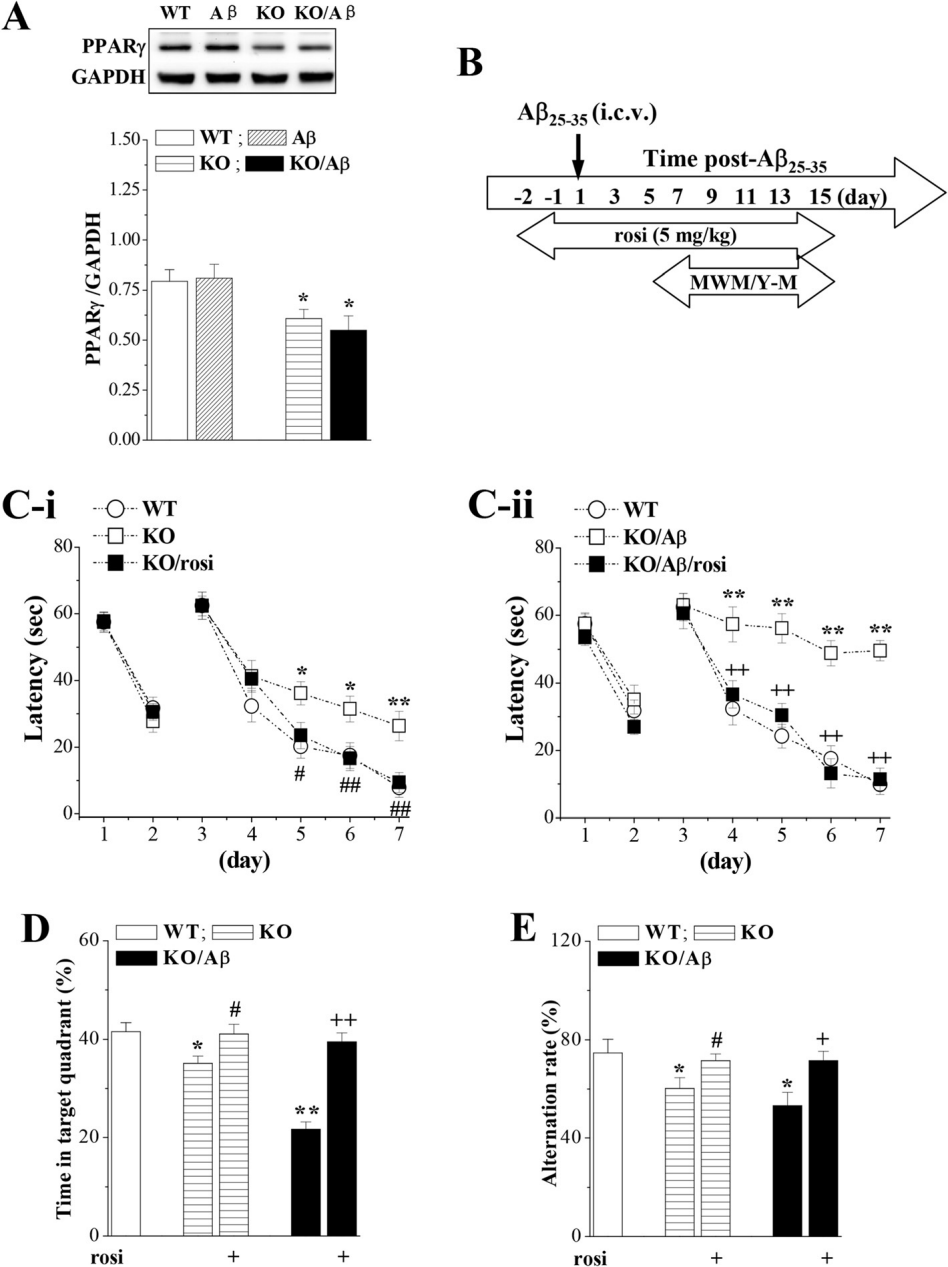
Activation of PPARγ prevents enhanced Aβ_25–35_-induced cognitive deficits by seipin deficiency. **a** The *bars* indicate the densitometric values of the hippocampal PPARγ protein in WT mice (WT), Aβ_25–35_ mice (Aβ), *seipin*-KO mice (KO), and Aβ_25–35_-KO mice (KO/Aβ). *P < 0.05 vs. WT mice (two-way ANOVA with Bonferroni’s test). **b** Time chart of the experimental procedure. *MWM* Morris water maze task, *Y-M* Y-maze task. **c** *-i* and **c-** *ii* Each point represents the group mean latency (s) to reach the visible and hidden platforms in WT mice, vehicle-treated *seipin*-KO mice (KO), rosi-treated *seipin*-KO mice (KO/rosi), Aβ_25–35_-KO mice (KO/Aβ), and Aβ_25–35_-KO mice (KO/Aβ/rosi). *P < 0.05 and **P < 0.01 vs. WT mice; ^#^ *P* < 0.05 and ^##^ *P* < 0.01 vs. *seipin*-KO mice; ^++^ *P* < 0.01 vs. KO/Aβ mice (repeated-measures ANOVA with Bonferroni’s test). **d** The *bars* represent the percentages of time spent in a target quadrant of the probe test. *P < 0.05 and **P < 0.01 vs. WT mice; ^#^ *P* < 0.05 vs. *seipin*-KO mice; ^++^ *P* < 0.01 vs. KO/Aβ mice (two-way ANOVA with Bonferroni’s test). **e** *Bar graph* indicates the alternation rates (%) in the Y-maze task. *P < 0.05 vs. WT mice; ^#^ *P* < 0.05 vs. *seipin*-KO mice; ^+^ *P* < 0.05 vs. KO/Aβ mice (two-way ANOVA with Bonferroni’s test)

The level of hippocampal PPARγ exhibited a main effect of genotype (*F*_(1, 28)_ = 12.778, P < 0.001; Fig. 2a) but no effect of Aβ_25–35_ (*F*_(1, 28)_ = 0.037, P = 0.848) or genotype × Aβ_25–35_ interaction (*F*_(1, 28)_ = 0.374, P = 0.546). Compared with the WT mice, the level of hippocampal PPARγ was remarkably reduced in the *seipin*-KO mice (*P* < 0.05) but was not significantly different compared with that in the Aβ_25–35_-KO mice (*P* > 0.05).

Consistent with the report by Zhou et al. [7], the treatment of the *seipin*-KO mice with the PPARγ agonist rosiglitazone (rosi, 5 mg/kg, p.o.) for consecutive 17 days corrected the prolongation of escape latencies to reach the hidden platform (*P* < 0.05, *n* = 16; Fig. 2c-*i*) and the decrease in the swimming time of target quadrant (*P* < 0.05, *n* = 16; Fig. 2d) and the alternation ratio of Y-maze (KO: *P* < 0.05, *n* = 8; Aβ_25–35_*-*KO: *P* < 0.05, *n* = 8; Fig. 2e). To test the involvement of the reduced PPARγ level in the Aβ_25–35_-induced cognitive deficits, the rosi treatment was given for consecutive 17 days starting from 2 days before the Aβ_25–35_ injection (Fig. 2b). In comparison with the vehicle-treated Aβ_25–35_*-*KO mice, the rosi treatment in the Aβ_25–35_*-*KO mice could perfectly reduce their escape latencies reaching the hidden platform (*P* < 0.01, *n* = 16; Fig. 2c-*ii*) and increase the swimming time in the target quadrant (*P* < 0.01, *n* = 16) and alternation ratio in the Y-maze (*P* < 0.05, *n* = 8).

### Seipin deficiency enhances Aβ_25–35_-induced death of neuronal cells via PPARγ reduction

**Fig. 3:**
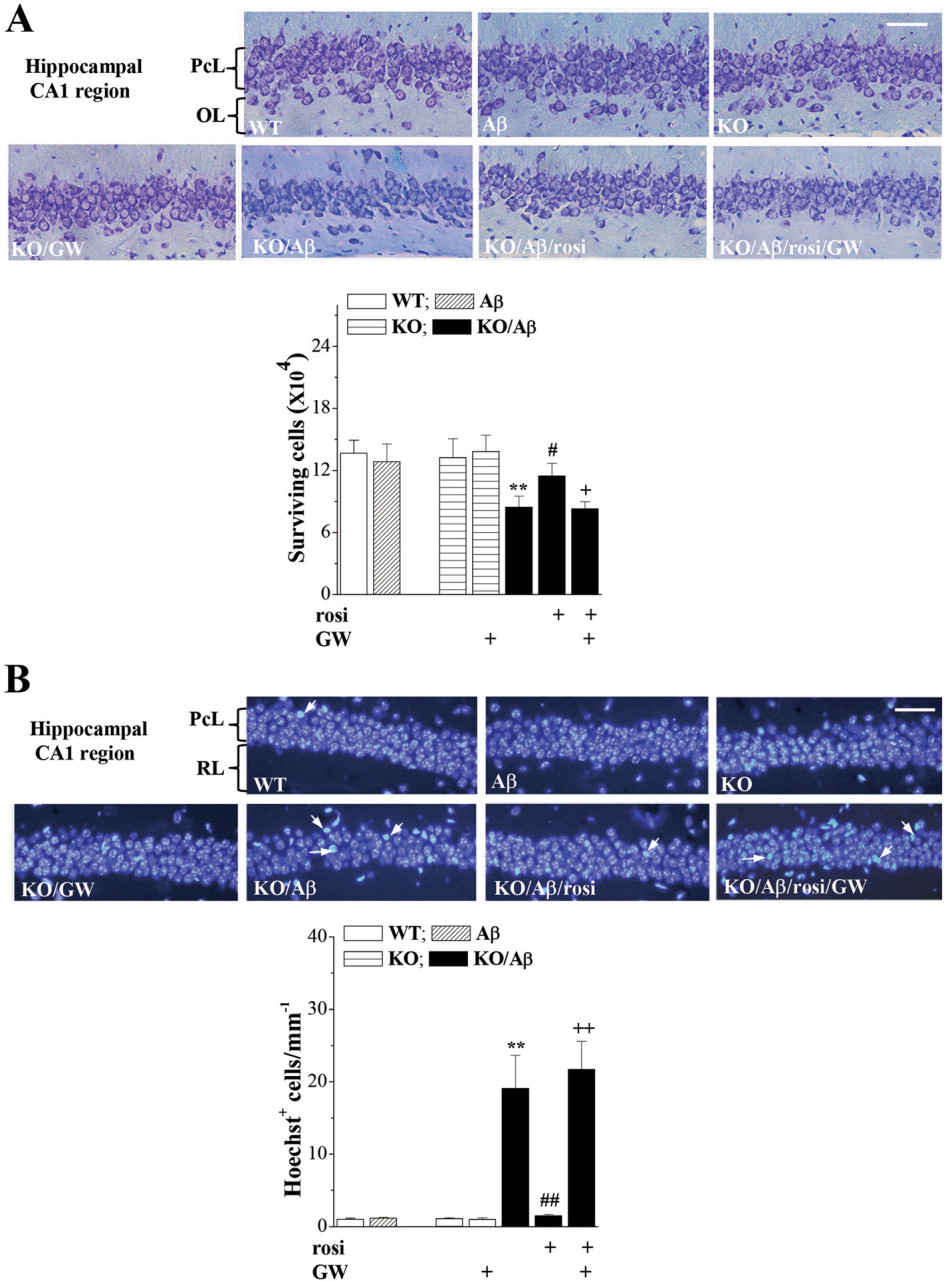
Activation of PPARγ attenuates enhanced Aβ neurotoxicity by seipin deficiency. **a** Representative images of the hippocampal CA1 regions (5-μm sections stained with toluidine blue) in WT mice (WT), Aβ_25–35_ mice (Aβ), *seipin*-KO mice (KO), Aβ_25–35_-KO mice (KO/Aβ), rosi-treated Aβ_25–35_-KO mice (KO/Aβ/rosi), GW9662-treated *seipin*-KO mice (KO/GW), and KO/Aβ/rosi mice (KO/Aβ/rosi/GW). *PcL* pyramidal cell layer, *OL* oriens layer. *Scale bars* = 50 μm. The *bar graph* represents the stereological counts of the surviving pyramidal cells. **P < 0.01 vs. WT mice; ^#^ *P* < 0.05 vs. Aβ_25–35_-KO mice; ^+^ *P* < 0.05 vs. KO/Aβ/rosi mice (three-way ANOVA with Bonferroni’s test). **b** Representative images of Hoechst staining in the hippocampal CA1 regions. The *white arrows* indicate the Hoechst^+^ cells. PcL: pyramidal cell layer; RL: radiatum layer. Scale bars = 50 μm. The bars indicate the density of Hoechst^+^ cells. **P < 0.01 vs. WT mice; ^##^ *P* < 0.01 vs. Aβ_25–35_-KO mice; ^++^ *P* < 0.01 vs. KO/Aβ/rosi mice (three-way ANOVA with Bonferroni’s test)

There was no main effect of genotype (*F*_(1, 28)_ = 1.572, *P* = 0.220; Fig. 3a) or Aβ_25–35_ (*F*_(1, 28)_ = 2.091, *P* = 0.159) on the number of pyramidal cells in the hippocampal CA1 region; however, there was a significant genotype × Aβ_25–35_ interaction (*F*_(1, 28)_ = 4.294, *P* = 0.048). Stereological counts of the surviving pyramidal cells revealed an approximately 35 % loss of pyramidal cells in the Aβ_25–35_*-*KO mice (*P* < 0.01) and no loss in the Aβ_25–35_-mice (*P* > 0.05). As illustrated in Fig. 3b, large numbers of Hoechst-positive (Hoechst^+^) cells were observed in the Aβ_25–35_-KO mice (*P* < 0.01, *n* = 8) but not in the *seipin*-KO mice (*P* > 0.05, *n* = 8) or the Aβ_25–35_-mice (*P* > 0.05, *n* = 8). The rosi treatment in the Aβ_25–35_-KO mice could attenuate the deaths of pyramidal cells (*P* < 0.05) and the number of Hoechst^+^ cells (*P* < 0.01), which were sensitive to the PPARγ antagonist GW9962 (pyramidal cells: *P* < 0.05; Hoechst^+^ cells: *P* < 0.01). The administration of GW9962 alone in the *seipin*-KO mice did not affect the survival of pyramidal cells (*P* > 0.05). Similarly, the Aβ_1–42_ injection in the *seipin*-KO mice rather than the WT mice caused approximately 30 % death of pyramidal cells (*P* < 0.05, *n* = 8; Additional file 1: Figure S1), which was prevented by the rosi treatment (*P* < 0.05, *n* = 8).

### Seipin deficiency facilitates Aβ_25–35_-induced inflammation via PPARγ reduction

**Fig. 4:**
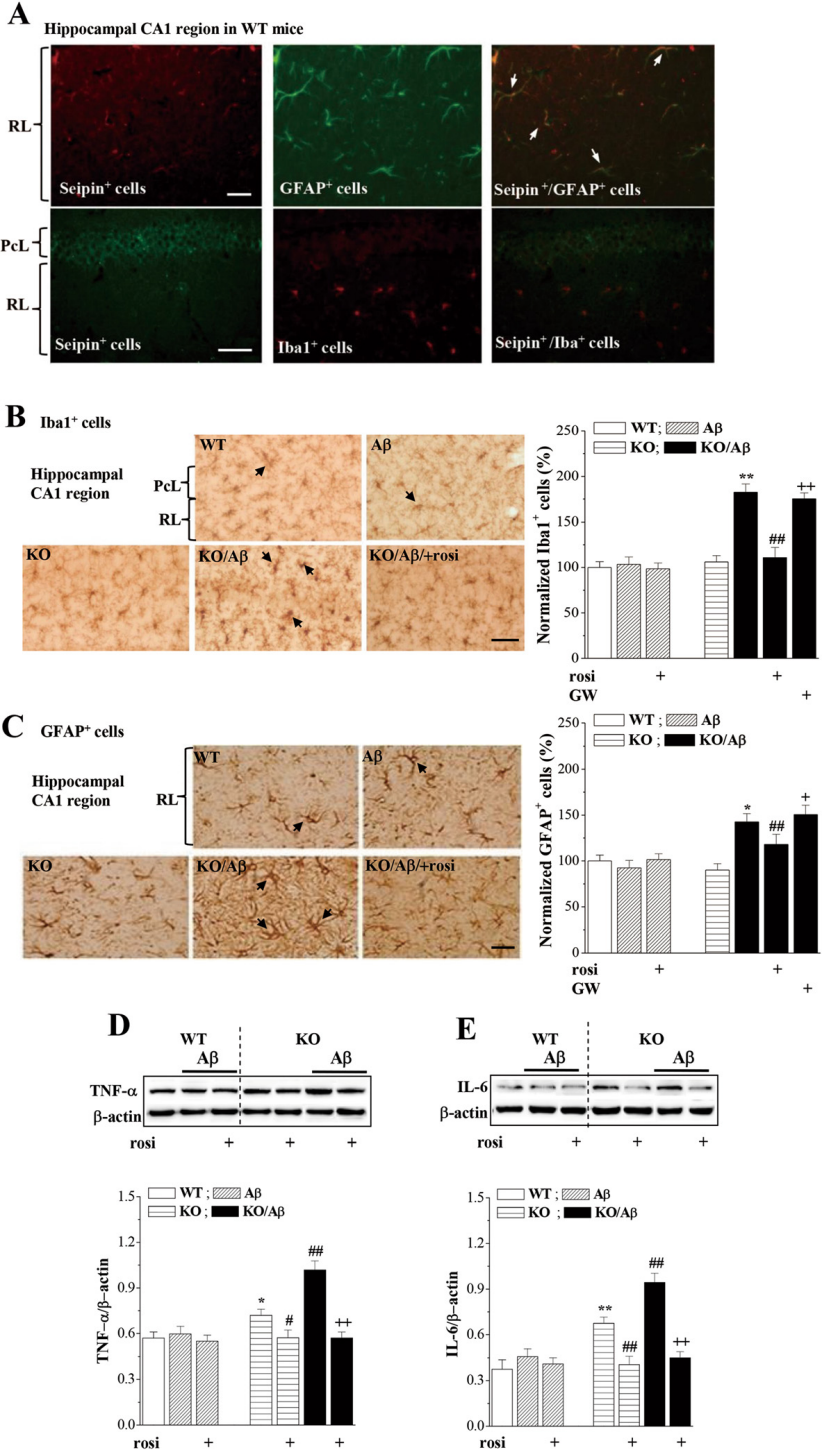
Activation of PPARγ reduces the enhanced Aβ-induced inflammation by seipin deficiency. **a** The upper panel shows representative fluorescence images by seipin (*red color*, *left column*) and GFAP (*green color*, *middle column*) double immuno-staining in hippocampal CA1 regions of WT mice (*scale bar* = 50 μm). The *white arrows* indicate seipin/GFAP-positive cells (*yellow color*, *right column*). The *bottom panels* shows the representative pictures of seipin (*green color*, *left column*) and Iba1 (*red color*, *middle column*) double immuno-staining in hippocampal CA1 regions of WT mice (*scale bar* = 50 μm). *PcL* pyramidal cell layer, *RL* radiatum layer. **b**, **c** Representative pictures of Iba1^+^ cells (*scale bar* = 50 μm) and GFAP^+^ cells (*scale bar* = 25 μm) in the hippocampal CA1 regions of WT mice (WT), Aβ_25–35_ mice (Aβ), *seipin*-KO mice (KO), Aβ_25–35_-KO mice (KO/Aβ), and rosi-treated KO/Aβ mice (KO/Aβ/rosi). The *bar graphs* represent the densities of Iba1^+^ microglial and GFAP^+^-activated astrocytes normalized by the control value obtained from WT mice. ^**^ *P* < 0.01 vs. *seipin*-KO mice; ^#^ *P* < 0.05 and ^##^ *P* < 0.01 vs. Aβ_25–35_-KO mice; ^+^ *P* < 0.05 and ^++^ *P* < 0.01 vs. KO/Aβ/rosi mice (three-way ANOVA with Bonferroni’s test). **d**, **e** Levels of hippocampal TNF-α and IL-1β. *P < 0.05 and **P < 0.01 vs. WT mice; ^#^ *P* < 0.05 and ^##^ *P* < 0.01 vs. *seipin*-KO mice; ^++^ *P* < 0.01 vs. Aβ_25–35_-KO mice (three-way ANOVA with Bonferroni’s test)

Sequentially, we observed that GFAP^+^ astrocytes expressed highly the seipin protein in the hippocampal CA1 region (upper panels in Fig. 4a) but Iba1^+^ microglia did not (bottom panels). The numbers of Iba1^+^ cells (*P* > 0.05, *n* = 8; Fig. 4b) or GFAP^+^ cells (*P* > 0.05, *n* = 8; Fig. 4c) in the *seipin*-KO mice did not differ from those in the WT mice. Morphological analysis revealed GFAP^+^ stellated-shaped astrocytes with thin processes that denoted a resting phenotype or with thick processes that reflected an activated phenotype [40]. Notably, the numbers of Iba1^+^ cells (*P* < 0.01, *n* = 8) and GFAP^+^ activated astrocytes (*P* < 0.01, *n* = 8) were significantly increased in the Aβ_25–35_-KO mice compared with the *seipin*-KO mice, which were reduced by the rosi treatment (Iba1^+^: *P* < 0.01, *n* = 8; GFAP^+^: *P* < 0.05, *n* = 8). The inhibitory effects of rosi on the activation of Iba1^+^ cells (*P* < 0.01, *n* = 8) and GFAP^+^ activated astrocytes (*P* < 0.05, *n* = 8) in the Aβ_25–35_-KO mice could be blocked by the PPARγ antagonist GW9962. By contrast, Aβ_25–35_ mice did not show the changes in the numbers of Iba1^+^ cells (*P* > 0.05, *n* = 8) and GFAP^+^ cells (*P* > 0.05, *n* = 8) compared to the WT mice. Additionally, the Aβ_1–42_ injection could increase the numbers of Iba1^+^ cells (*P* < 0.01, *n* = 8; Additional file 1: Figure S2) and GFAP^+^ activated astrocytes (*P* < 0.01, *n* = 8) in the *seipin*-KO mice but not in the WT mice, which was sensitive to the rosi treatment (Iba1^+^ cells: *P* < 0.01, *n* = 8; GFAP^+^ cells: *P* < 0.05, *n* = 8).

Further experiment was designed to examine the influence of seipin deficiency on Aβ-induced inflammatory reactions using Western blot. Notably, there were a main effect of genotype for the levels of hippocampal TNF-α (*F*_(1, 28)_ = 29.253, *P* < 0.001; Fig. 4d) and IL-6 (*F*_(1, 28)_ = 65.073, *P* < 0.001; Fig. 4e). Additionally, there was a significant genotype × Aβ_25–35_ interaction, because the levels of TNF-α (*P* < 0.01) and IL-6 (*P* < 0.01) were higher in the Aβ_25–35_-KO mice than those in the *seipin*-KO mice. However, the levels of TNF-α (*P* > 0.05) and IL-6 (*P* > 0.05) exhibited no differences between the WT mice and Aβ_25–35_ mice. Furthermore, the increased TNF-α and IL-6 levels in the *seipin*-KO mice (TNF-α: *P* < 0.05; IL-6: *P* < 0.01) and the Aβ_25–35_-KO mice (TNF-α: *P* < 0.01; IL-6: *P* < 0.01) were attenuated by the rosi treatment.

### Seipin deficiency increases GSK3β activity via PPARγ reduction

**Fig. 5:**
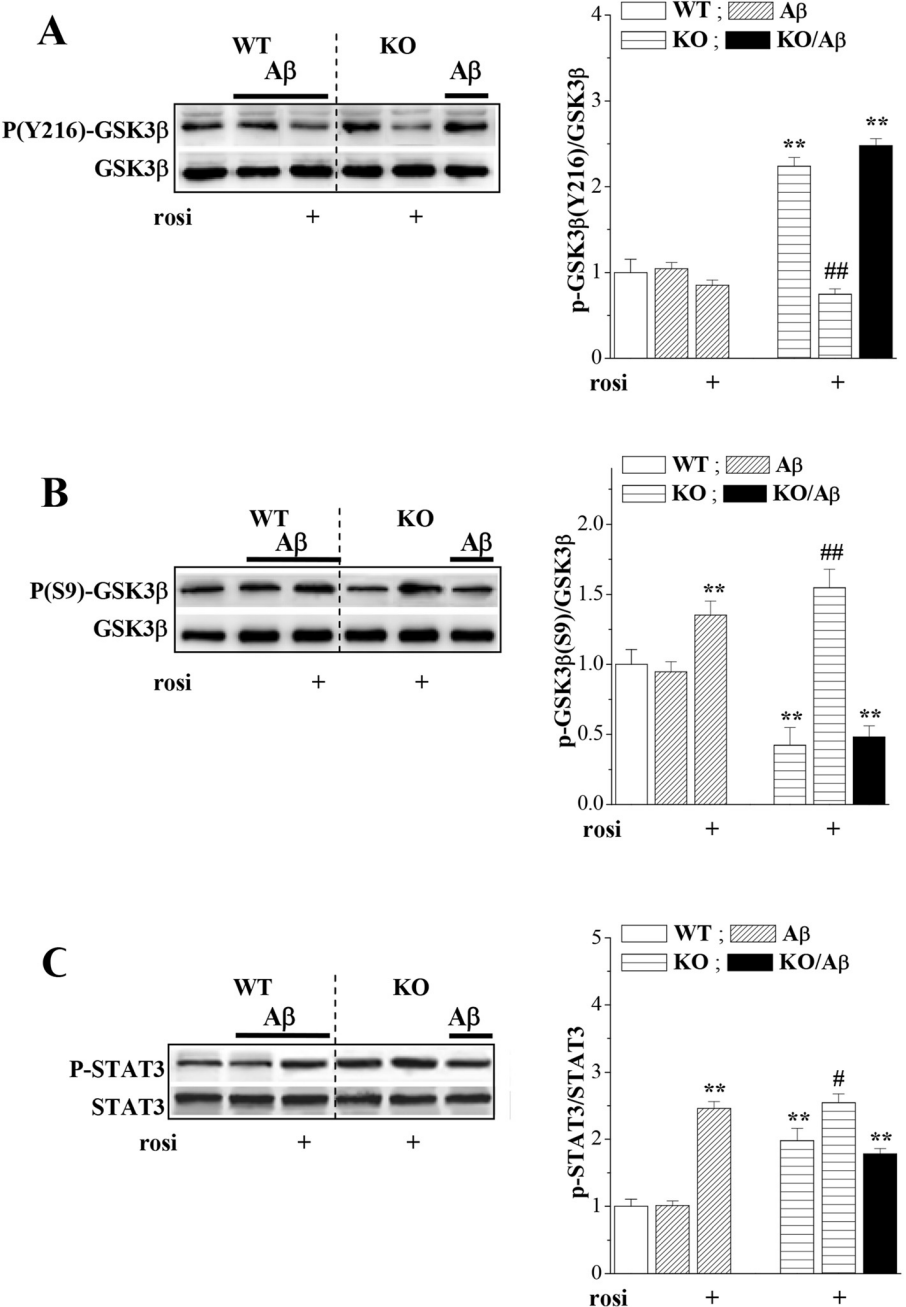
Activation of PPARγ depresses enhanced GSK3β activity by seipin deficiency. **a–c** Levels of hippocampal Tyr216 phospho-GSK3β, Ser9 phospho-GSK3β, and phospho-STAT3 in WT mice (WT), Aβ_25–35_ mice (Aβ), *seipin*-KO mice (KO), Aβ_25–35_-KO mice (KO/Aβ), and rosi-treated Aβ mice and KO/Aβ mice. Levels of Tyr216 phospho-GSK3β and Ser9 phospho-GSK3β were first normalized to the amount of GSK3β protein and subsequently normalized to the basal values in WT mice. **P < 0.01 vs. WT mice; ^##^ *P* < 0.01 vs. Aβ_25–35_-KO mice (three-way ANOVA). The levels of phospho-STAT3 were normalized to the amount of STAT3 protein and subsequently normalized to the basal values in WT mice. **P < 0.01 vs. WT mice; ^#^ *P* < 0.05 vs. Aβ_25–35_-KO mice (three-way ANOVA with Bonferroni’s test)

GSK3β has been reported to modulate pro-inflammatory cytokines [26]. GSK3β activity is negatively and positively regulated by phosphorylation at Ser9 and Tyr216, respectively. To explore the mechanisms underlying the seipin deficiency-increased TNF-α and IL-6, we examined the levels of hippocampal GSK3β phosphorylation (phospho-GSK3β) at Tyr216 and Ser9 [41]. Compared with the WT mice, the level of Tyr216 phospho-GSK3β was elevated (*P* < 0.01, *n* = 8; Fig. 5a) and the level of Ser9 phospho-GSK3β was reduced in the *seipin*-KO mice (*P* < 0.01, *n* = 8; Fig. 5b); and these levels were not affected by the Aβ_25–35_ injection. Interestingly, the rosi treatment in the *seipin*-KO mice obviously corrected the elevation of Tyr216 phospho-GSK3β (*P* < 0.01, *n* = 8) and the reduction of Ser9 phospho-GSK3β (*P* < 0.01, *n* = 8). Additionally, the rosi treatment of the WT mice increased Ser9 phospho-GSK3β (*P* < 0.01, *n* = 8) but had no effect on Tyr216 phospho-GSK3β (*P* > 0.05, *n* = 8).

The activation of GSK3β is required to activate tyrosine phosphorylation of STAT3 in astrocytes and microglia [24]. Similarly, the level of hippocampal STAT phosphorylation (phospho-STAT) in the *seipin*-KO mice was elevated compared with that in the WT mice (*P* < 0.01, *n* = 8; Fig. 5c). The level of phospho-STAT3 in the *seipin*-KO mice was further increased by the rosi treatment (*P* < 0.01, *n* = 8) but was not affected by the Aβ_25–35_ injection (*P* > 0.05, *n* = 8). Additionally, the rosi treatment increased the level of phospho-STAT3 in the WT mice (*P* < 0.05, *n* = 8).

## Discussion

The present study provides, for the first time, in vivo evidence that the seipin deficiency in astrocytes can facilitate the Aβ_25–35/1–42_-induced neuroinflammation to aggravate the Aβ_25–35/1–42_ neurotoxicity. This conclusion was deduced mainly from the following observations: (1) seipin was highly expressed in hippocampal pyramidal cells and astrocytes; (2) the seipin deficiency did not alter the number of neuronal cells, astrocytes, and microglia, but the levels of IL-6/TNF-α were higher in the *seipin*-KO mice than those in WT mice; (3) the injection (i.c.v.) of Aβ_25–35_ (1.2 nmol/mouse) or Aβ_1–42_ (0.1 nmol/mouse) could stimulate the activation of astrocytes and microglia and further increased the expression of IL-6/TNF-α in *seipin*-KO mice, but not in the WT mice; (4) the injection (i.c.v.) of Aβ_25–35_ or Aβ_1–42_, at the non-neurotoxic doses in WT mice, caused approximately 30–35 % death of pyramidal cells with a significant increase of apoptotic cells in hippocampal CA1 of the *seipin*-KO mice leading to the impairment of spatial memory.

### Seipin deficiency in astrocytes facilitates Aβ-neuroinflammation via the reduction of PPARγ

The treatment of astrocytes and microglia with Aβ_1–42_ can increase the production of IL-6 and TNF-α, which is inhibited by the activation of PPARγ [42]. The PPARγ agonists can prevent the overproduction of IL-6 and TNF-α in astrocytes and microglia treated with lipopolysaccharide (LPS) [43]. The injection (i.c.v.) of Aβ_25–35_ stimulates microglial activation in the brains of C57BL/6J mice [44]. The injection (i.c.v.) of Aβ_25–35_ (1.2 nmol/mouse) or Aβ_1–42_ (0.1 nmol/mouse) was insufficient to stimulate the activation of astrocytes and microglia or to increase the production of TNF-α or IL-6 in WT mice. However, this injection (i.c.v.) of Aβ_25–35_ or Aβ_1–42_ in the *seipin*-KO mice caused the activation of astrocytes and microglia with an obvious increase in the level of TNF-α or IL-6. Interestingly, in particular, the activation of PPARγ in the *seipin*-KO mice was able to prevent the Aβ_25–35/1–42_-induced neuroinflammation. Similarly, PPARγ-deficient heterozygous mice develop an exacerbated experimental allergic encephalomyelitis (EAE) in comparison with the wild-type littermates [15]. The exacerbation of EAE by the PPARγ antagonists associates with an augmented neural inflammation [16]. Astrocytes and microglia are fundamental cellular targets for the beneficial effects of PPARγ ligands [13]. We observed the seipin expression in hippocampal astrocytes. Although the number of astrocytes in the hippocampal CA1 region of the *seipin*-KO mice did not differ in comparison with the WT mice, the levels of hippocampal TNF-α and IL-6 in the *seipin*-KO mice were higher than those in the WT mice. In particular, the elevation of TNF-α or IL-6 in the *seipin*-KO mice was reduced by the PPARγ agonist. Thus, it may be possible that the reduction of PPARγ leads to the overproduction of pro-inflammatory cytokines in the *seipin*-KO mice, which facilitates the Aβ_25–35/1–42_-induced neuroinflammation. Another indirect but important supporting data is that the increased TNF-α and IL-6 in the *seipin*-KO mice could be corrected by the activation of PPARγ. Therefore, the findings give an indication that the seipin deficiency in astrocytes is able to facilitate the Aβ-induced neuroinflammation probably through reducing PPARγ to elevate the levels of TNF-α and IL-6.

### Seipin deficiency enhances GSK3β activity to increase pro-inflammatory cytokines

GSK3β has been identified as a strong promoter of pro-inflammatory cytokines, including IL-6 and TNF-α [25, 26]. The inhibition of GSK3β increases inflammatory tolerance and reduces inflammatory sensitization in the brain [45]. GSK3β inhibition in glial cells reduces pro-inflammatory responses by blocking STAT3 signaling [46]. The inhibition of GSK3 reduces STAT3 activation, IL-6 production, and GFAP up-regulation by LPS-stimulated primary glia [47]. The activation of PPARγ potentially suppresses the activator of STAT [48]. The level of STAT3 phosphorylation was significantly increased in the *seipin*-KO mice [10]. STAT3 activation is highly dependent on GSK3β in mouse primary astrocytes [24]. Notably, the catalytic activity of GSK3β in *seipin*-KO mice was enhanced as indicated by the elevation of Tyr216 phospho-GSK3β and the reduction of Ser9 phospho-GSK3β. The activation of PPARγ regulates negatively the expressions of GSK3β [49], but the level of GSK3β protein failed to be increased in the *seipin*-KO mice. Strangely enough, the administration of rosi to the *seipin*-KO mice could suppress the increase of GSK3β activity but further enhanced the phosphorylation of STAT3. Therefore, it is indicated that seipin deficiency in astrocytes through reducing PPARγ enhances the GSK3β activity rather than the STAT3 signaling to increase the production of TNF-α and IL-6. On the other hand, GSK3β regulates interferon-γ (IFN-γ) signaling and is involved in IFN-γ-induced inflammation [50]. Thus, further studies are required to examine whether the GSK3β activity in *seipin*-KO mice through synergistically facilitating IFN-γ-induced STAT1 activation increases the production of TNF-α [41].

### Seipin deficiency enhances Aβ-neurotoxicity via the reduction of PPARγ

A principal finding in this study is that seipin deficiency in hippocampal neuronal cells enhances the Aβ_25–35/1–42_ neurotoxicity as indicated by a massive death of pyramidal cells in the *seipin*-KO mice treated with the Aβ_25–35_ (1.2 nmol/mouse) or Aβ_1–42_ (0.1 nmol/mouse), generally non-neurotoxic doses in the WT mice or control mice [29]. The level of the hippocampal PPARγ was significantly reduced in the *seipin*-KO mice. The decline of PPARγ in the *seipin*-KO mice [11] or the neuronal specific PPARγ knockout in mice [51] did not cause the loss of neuronal cells in the hippocampus. The rosi treatment could significantly reduce the Aβ_25–35/1–42_-induced death of pyramidal cells in the *seipin*-KO mice. Activated microglia and reactive astrocytes can produce cytokines, reactive oxygen species, and other neurotoxic substances to cause neuronal apoptosis and other series of pathologic events. The progressive neuroinflammation and neuronal apoptosis in AD is considered to be a consequence of the Aβ-neurotoxic properties [52, 53]. Our data support the notion that Aβ_25–35/1–42_-induced neuroinflammation in the *seipin*-KO mice causes the death of neuronal cells. Zhao et al. [51] reported that neuronal PPARγ deficiency increased susceptibility to brain damage after cerebral ischemia through suppressing the expressions of Cu-Zn superoxide dismutase (SOD1), catalase (CAT), and glutathione S-transferase (GST). However, the levels of hippocampal *SOD1*, *CAT*, and *GST* expression in *seipin*-KO mice did not differ from WT mice (data not shown). Additionally, Ito et al. reported that mutations (N88S/S90L) of the seipin gene can cause the formation of cytoplasmic inclusions and enhance ubiquitination, which leads to endoplasmic reticulum (ER) stress [54]. However, this idea is not supported because the levels of the ER stress makers BiP and CHOP in *seipin*-KO mice are not increased [11].

## Conclusions

Using the *seipin*-KO mice with reduction of PPARγ, we observed that seipin deficiency elevated the activity of GSK3β to enhance the production of TNF-α and IL-6, which in turn triggered and strengthened the Aβ_25–35_-induced inflammatory responses leading to the death of neuronal cells and the spatial cognitive deficits. This is particularly true of neuroinflammation, which contributes to a broad range of neurodegenerative diseases [55]. Although much more work needs to be performed in the future, this is the first report to demonstrate that the expression of seipin in hippocampal astrocytes is required for regulating the inflammatory responses and preventing the neurodegeneration.

## Highlights

1. Hippocampal GSK3β activity and IL-6/TNF-α were slightly increased in seipin-KO mice.
2. PPARγ agonist inhibits increased GSK3β activity and IL-6/TNF-α in KO mice.
3. Low-dose Aβ_25–35_ causes death of pyramidal cells in KO mice, not in WT mice.
4. Aβ_25–35_ activates microglia and astrocytes to increases IL-6/TNF-α in KO mice.
5. PPARγ agonist prevents Aβ_25–35_-neuroinflammation and -neurotoxicity in KO mice.

## Abbreviations

AD, Alzheimer’s disease; AGPAT2, 1-acylglycerol-3-phosphate-O-acyl transferase 2; Aβ, β-amyloid; BSCL2, Berardinelli-Seip congenital lipodystrophy 2; CGL, congenital generalized lipodystrophy; GFAP, glial fibrillary acidic protein; GSK3, glycogen synthase kinase-3; i.c.v., intracerebroventricular; Iba1^+^, ionized calcium-binding adaptor molecule 1-positive; IL-6, interleukin-6; PA, phosphatidic acid; PPARγ, peroxisome proliferator-activated receptor gamma; *seipin*-KO mice, seipin knockout mice; STAT, signal transducer and activator of transcription; TNF-α, tumor necrosis factor-α

## Additional file

Additional file 1: Effects of neuronal seipin knockout on Aβ_1-42_-induced neurotoxicity and neuroinflammation. (116 KB)
