# Supplementary material for: Neuronal seipin knockout facilitates Aβ-induced neuroinflammation and neurotoxicity via reduction of PPARγ in hippocampus of mouse
Source: J Neuroinflammation. 2016 Jun 10;13:145. doi: 10.1186/s12974-016-0598-3 (PMC4902906; doi:10.1186/s12974-016-0598-3)
Supplement: Additional file 1: — Effects of neuronal seipin knockout on Aβ1-42-induced neurotoxicity and neuroinflammation. (116 KB) [file 12974_2016_598_MOESM1_ESM.doc]

***Additional files***

**Effects of neuronal seipin knockout on Aβ_1-42_-induced neurotoxicity and neuroinflammation**

**[Additional file 1](http://eproofing.springer.com/journals/mainpage.php?token=1u0PtX3yOTW2btzUZ5qSfymyvU_Q9pDN#AQ7)**

**Fig. S1** Activation of PPARγ attenuates the Aβ_1-42_-neurotoxicity in *seipin*-KO mice. Bar graphs represent the numbers of surviving pyramidal cells in the hippocampal CA1 regions of WT mice (WT), Aβ_1-42_-mice (Aβ_1-42_), *seipin*-KO mice (KO), Aβ_1-42_-KO mice (KO/Aβ_1-42_). **P*<0.05 *vs*. *seipin*-KO mice; #*P*<0.05 *vs*. KO/Aβ_1-42_ mice.

**Additional file 2**

**Fig. S2** Activation of PPARγ prevents the Aβ_1-42_-induced inflammation in *seipin*-KO mice. Bar graphs represent the numbers of Iba1+ microglial (**a**) and GFAP+ activated astrocytes (**b**) in the hippocampal CA1 regions of WT mice (WT), Aβ_1-42_-mice (Aβ_1-42_), *seipin*-KO mice (KO), Aβ_1-42_-KO mice (KO/Aβ_1-42_). ##*P*<0.01 *vs*. *seipin*-KO mice; ++*P*<0.01 and +*P*<0.05 *vs*. KO/Aβ_1-42_ mice.
